# Myostatin inhibition in combination with antisense oligonucleotide therapy improves outcomes in spinal muscular atrophy

**DOI:** 10.1002/jcsm.12542

**Published:** 2020-02-07

**Authors:** Haiyan Zhou, Jinhong Meng, Alberto Malerba, Francesco Catapano, Palittiya Sintusek, Susan Jarmin, Lucy Feng, Ngoc Lu‐Nguyen, Lianwen Sun, Virginie Mariot, Julie Dumonceaux, Jennifer E. Morgan, Paul Gissen, George Dickson, Francesco Muntoni

**Affiliations:** ^1^ Genetics and Genomic Medicine Research and Teaching Department, Great Ormond Street Institute of Child Health University College London London UK; ^2^ The Dubowitz Neuromuscular Centre, Developmental Neurosciences Research and Teaching Department, Great Ormond Street Institute of Child Health University College London London UK; ^3^ Centres of Gene and Cell Therapy and Biomedical Sciences, School of Biological Sciences Royal Holloway, University of London Egham UK; ^4^ Department of Paediatrics, Faculty of Medicine, King Chulalongkorn Memorial Hospital Chulalongkorn University Bangkok Thailand; ^5^ Beijing Advanced Innovation Centre for Biomedical Engineering Beihang University Beijing China; ^6^ NIHR Great Ormond Street Hospital Biomedical Research Centre London UK

**Keywords:** Spinal muscular atrophy, Survival of motor neuron 1 gene, Antisense oligonucleotide, Myostatin inhibition, Adeno‐associated virus, Myostatin propeptide, Combinatorial therapy

## Abstract

**Background:**

Spinal muscular atrophy (SMA) is caused by genetic defects in the *survival motor neuron 1* (*SMN1*) gene that lead to SMN deficiency. Different SMN‐restoring therapies substantially prolong survival and function in transgenic mice of SMA. However, these therapies do not entirely prevent muscle atrophy and restore function completely. To further improve the outcome, we explored the potential of a combinatorial therapy by modulating SMN production and muscle‐enhancing approach as a novel therapeutic strategy for SMA.

**Methods:**

The experiments were performed in a mouse model of severe SMA. A previously reported 25‐mer morpholino antisense oligomer PMO25 was used to restore SMN expression. The adeno‐associated virus‐mediated expression of myostatin propeptide was used to block the myostatin pathway. Newborn SMA mice were treated with a single subcutaneous injection of 40 μg/g (therapeutic dose) or 10 μg/g (low‐dose) PMO25 on its own or together with systemic delivery of a single dose of adeno‐associated virus‐mediated expression of myostatin propeptide. The multiple effects of myostatin inhibition on survival, skeletal muscle phenotype, motor function, neuromuscular junction maturation, and proprioceptive afferences were evaluated.

**Results:**

We show that myostatin inhibition acts synergistically with SMN‐restoring antisense therapy in SMA mice treated with the higher therapeutic dose PMO25 (40 μg/g), by increasing not only body weight (21% increase in male mice at Day 40), muscle mass (38% increase), and fibre size (35% increase in tibialis anterior muscle in 3 month female SMA mice), but also motor function and physical performance as measured in hanging wire test (two‐fold increase in time score) and treadmill exercise test (two‐fold increase in running distance). In SMA mice treated with low‐dose PMO25 (10 μg/g), the early application of myostatin inhibition prolongs survival (40% increase), improves neuromuscular junction maturation (50% increase) and innervation (30% increase), and increases both the size of sensory neurons in dorsal root ganglia (60% increase) and the preservation of proprioceptive synapses in the spinal cord (30% increase).

**Conclusions:**

These data suggest that myostatin inhibition, in addition to the well‐known effect on muscle mass, can also positively influence the sensory neural circuits that may enhance motor neurons function. While the availability of the antisense drug Spinraza for SMA and other SMN‐enhancing therapies has provided unprecedented improvement in SMA patients, there are still unmet needs in these patients. Our study provides further rationale for considering myostatin inhibitors as a therapeutic intervention in SMA patients, in combination with SMN‐restoring drugs.

## Introduction

Spinal muscular atrophy (SMA) is a severe neuromuscular disease caused by loss‐of‐function mutations in the *survival of motor neuron 1* (*SMN1*) gene, which encodes the SMN protein.^1^ Its neighbouring centromeric *SMN2* gene is intact in all patients but cannot fully compensate for the loss of *SMN1*, due to the alternative splicing and exclusion of exon 7 from most of its transcript.^2^, ^3^

Experimental therapies, including antisense oligonucleotide (AON) therapy,^4^, ^5^, ^6^, ^7^ adeno‐associated virus (AAV) mediated SMN1 gene therapy,^8^, ^9^, ^10^ and small molecule therapy,^11^ have achieved promising results in preclinical studies and clinical trials.^12^ Spinraza™ (Nusinersen) is the first Food and Drug Administration and European Medicines Agency approved antisense drug for treatment in SMA.^13^, ^14^ We and others have shown that while SMN‐restoring AONs can strikingly prolong the survival of SMA mice, the body mass and muscle phenotypes can only be partially rescued.^4^, ^6^ Similar observations were also made in the SMN1 gene therapy and small molecule therapies.^8^, ^11^ We therefore explored a new therapeutic strategy to ameliorate the partially rescued skeletal muscle phenotype, which could be used synergistically with one of the existing SMN‐restoring therapies.

Myostatin is a member of the transforming growth factor β (TGF‐β) superfamily. It is synthesized and expressed predominantly in skeletal muscle and negatively regulates skeletal muscle growth.^15^, ^16^, ^17^ Circulating myostatin is bound to at least two major proteins, myostatin propeptide (MPRO) and follistatin, to form a complex.^18^ The binding of MPRO and follistatin to myostatin inhibits its activity. Myostatin must be cleaved from its complex by metalloproteinase to generate an active ligand that can then bind with the type IIB activin receptor (ActRIIB). The binding of myostatin and ActRIIB initiates a series of intracellular signalling cascades and transcriptional activation of target genes such as myogenic regulatory factors that eventually regulate skeletal muscle growth and differentiation.^16^, ^19^ Suppression of myostatin activity and signalling pathway by different strategies have been used to increase skeletal muscle mass.^16^, ^20^, ^21^, ^22^ These strategies include myostatin‐neutralizing antibodies, antibodies directed against the myostatin receptor (ActRIIB), the use of AONs to induce out‐of‐frame exon‐skipping and nonsense‐mediated mRNA decay, ligand traps such as ActRIIB‐Fc fusion proteins, and viral vectors to force expression of myostatin inhibitors (MPRO and follistatin).^16^, ^20^, ^21^, ^22^ Myostatin inhibition has been investigated as a therapeutic strategy in Duchenne muscular dystrophy (DMD) with encouraging outcomes in preclinical studies.^23^, ^24^ In the DMD mouse model, the delivery of myostatin propeptide gene by AAV vectors (AAV‐MPRO) significantly increased muscle growth and improved dystrophic phenotypes.^24^ However, the relevance of these findings for DMD patients has been questioned, following the failure to meet the clinical endpoint in a recent clinical trial using a myostatin‐neutralizing antibody (NCT02310763). This has been ascribed, at least partially, to lower levels of circulating myostatin in patients with DMD, and the lack of sufficient activation of this pathway to justify its further inhibition.^25^

Recent studies in SMA mice suggest that myostatin inhibition, together with an SMN‐restoring small molecule therapy, could be potentially beneficial.^26^, ^27^ A number of questions however remain unanswered: what is an optimal timing for considering myostatin inhibition in SMA? Is the mechanism of action simply related to the muscle hypertrophy? What is the extent of the functional improvement that can be expected from a combinatorial therapy where muscle‐enhancing and an SMN‐restoring strategy are considered?

In this study, we investigated the combinatorial therapy of SMN‐restoring antisense therapy with myostatin inhibition in the Taiwanese SMA mouse model.^28^ We used the previously reported 25‐mer morpholino antisense oligomer PMO25, which augments *SMN2* exon 7 splicing and restores SMN protein, to rescue the severe SMA mice.^6^ The systemic administration of myostatin propeptide gene by AAV vectors (AAV‐MPRO) was used to block the myostatin pathway, by preventing the binding of myostatin to its receptor ActRIIB, as previously reported in the DMD mouse model.^24^ To try to recapitulate the potential of an additive effect of myostatin inhibition in SMA patients with different disease severities and its potential effect in patients who may present different response to Spinraza treatment, we treated newborn SMA mice with either high (40 μg/g) or low (10 μg/g) dose of PMO25 by a single subcutaneous injection. We show in this study that myostatin inhibition acts synergistically with the SMN‐restoring AON. Myostatin inhibition improves the skeletal muscle phenotype and the physical performance in AON‐treated SMA mice. We also characterized in detail the effect of myostatin inhibition outside skeletal muscle, including neuromuscular junctions (NMJs), dorsal root ganglia (DRG), and proprioceptive synapses in the spinal cord. Our study provides further rationale for developing the combinatorial muscle‐enhancing and SMN‐restoring therapy for SMA.

## Methods

### Animals

Spinal muscular atrophy transgenic mice, FVB.Cg‐Tg (*SMN2*)_2_Hung *Smn1* ^tm1Hung^/J, were purchased from the Jackson Laboratory (TJL005058). All the procedures conducted in mice were carried out in the Biological Services Unit, University College London Great Ormond Street Institute of Child Health, in accordance with the Animals (Scientific Procedures) Act 1986. Experiments were performed under Home Office project licence PPL 70/8389.

### Morpholino antisense oligomer

PMO25 was synthesized by Gene Tools LLC. The sequence of PMO25 is GTA AGA TTC ACT TTC ATA ATG CTG G, complementary to 25 (‐10, ‐34) bases in the intron 7 of *SMN2* gene.^6^ PMO25 was made as a stock solution at a concentration of 20 μg/μL; and stored at room temperature. The concentration of the stock solution was determined by Nanodrop, according to the manufacturer's instructions. Newborn SMA mice received a single dose of PMO25 at either 40 or 10 μg/g as suggested by our previous studies.^6^, ^29^ PMO25 was injected in newborn SMA mice at postnatal day 0 (PND 0) by subcutaneous injection using a 10 μL glass capillary (Drummond Scientific Company).

### Adeno‐associated virus‐myostatin propeptide

The AAV8ProMyo vector was prepared using a standardized double transfection protocol. Briefly, the plasmid pProMyo was generated by cloning the myostatin propeptide sequence, under control of a CAG promoter, into a pDD‐derived AAV backbone.^30^ HEK293T cells were transfected with pProMyo and pAAV helper cap8 (pDF8 helper plasmid encoding viral cap and rep ORF) using polyethylenimine and cultured in Dulbecco's modified Eagle's medium with 2% fetal calf serum. Three days later, cells were lysated, and recombinant pseudotyped AAV vector particles (vp) were harvested and purified by iodixanol (Sigma‐Aldrich) step‐gradient (15–60%) ultracentrifugation (255 000× *g* for 90 min at 18 °C). The ioxidanol fraction containing the vector was collected and resuspended in PBS‐MK (phosphate buffered saline with 5 mM MgCl2 and 12.5 mM KCl). After desalting and concentrating using Amicon Ultra‐15 100,000K (PL100) (Millipore), the vector copy number was quantified by quantitative polymerase chain reaction (PCR). The titre of the AAV used for these experiments was 5 × 10^13^ vp/mL. A single dose of AAV‐MPRO at 1.67 × 10^10^ vp/g was injected subcutaneously in newborn SMA mice at PND 0, in order to have a body‐wide effect to all the skeletal muscles. The effect of AAV‐MPRO on skeletal muscle growth was measured by body weight gain and muscle mass described in the succeeding text.

### Real‐time polymerase chain reaction

Total RNA was extracted from tibialis anterior (TA) muscles in 10‐day‐old mice, and cDNA was synthesized using a SuperScript III Reverse Transcription Kit (Life Technologies). Quantitative real‐time PCR was performed with StepOne Real‐Time PCR Systems (Applied Biosystems) as described previously.^6^ The sequences of the primers for human‐specific full‐length *SMN2* (133 bp) are as follows: forward 5′‐ATA CTG GCT ATT ATA TGG GTT TT‐3′ and reverse 5′‐TCC AGA TCT GTC TGA TCG TTT C‐3′. The sequences of the primers for human‐specific Δ7 *SMN2* (125 bp) are as follows: forward 5′‐TGG ACC ACC AAT AAT TCC CC‐3′ and reverse 5′‐ATG CCA GCA TTT CCA TAT AAT AGC C‐3′. The sequences of the primers for mouse *Mstn* (97 bp) are as follows: forward 5′‐CAG GAG AAG ATG GGC TGA AT‐3′ and reverse 5′‐GAG TGC TCA TCG CAG TCA AG‐3′. Mouse *Gapdh* was used as reference gene.

### Histopathology and immunohistochemistry

Freshly dissected mouse TA muscles collected from 3‐month‐old mice were embedded in OCT (CellPath) on corks and frozen in liquid nitrogen‐cooled iso‐pentane. Transverse cryosections from muscles were cut at a thickness of 7 μm for haematoxylin and eosin and immunofluorescence staining. Muscle fibres were stained with rabbit polyclonal anti‐laminin primary antibody (L9393; 1:2000; Sigma‐Aldrich) to identify fibre boundaries. The staining of laminin was visualized with Alexa Fluor 488 goat anti‐rabbit IgG (H + L) (1:500; Life Technologies). Sections were mounted in Hydromount mounting medium (National Diagnostics). Images were digitally captured using Metamorph software. Approximately 500 myofibres from at least five different areas selected randomly from a representative section of each muscle were measured. The minimal Feret's diameter of myofibres were measured as recommended by TREAT‐NMD (http://www.treat‐nmd.org) and quantified using Image J software (http://imagej.nih.gov/ij/).

The spinal cord and DRG of lumbar segment collected from 20‐day‐old mice were post‐fixed in 4% paraformaldehyde and cryoprotected in 30% sucrose. Ten micrometre transverse sections were cut. Sections of the spinal cord were stained using antibodies against choline acetyltransferase (1:100; Millipore) and vesicular glutamate transporter 1 (vGLUT1) (1:100; Millipore). Sensory neurons in DRG were stained using vGLUT1 antibody. Sections were imaged using confocal scanning microscopy (Carl Zeiss LSM‐710). Motor neurons and vGLUT1^+^ synapses and sensory neurons were quantified from Z‐stack images using ImageJ software.

### Neuromuscular junction staining

Whole TA muscles collected from 20‐day‐old mice were fixed in 4% paraformaldehyde and permeabilized in 5% goat serum and 1% Triton X‐100 in PBS. Samples were then incubated in rabbit anti‐neurofilament (NF‐M, 1:100; Sigma) and synaptophysin (1:200; Synaptic Systems) antibodies overnight at 4 °C, followed by Alexa Fluor 488 goat anti‐rabbit IgG (H+L) (1:500; Life Technology) and rhodamine‐α‐bungarotoxin (α‐BT) (1:1000; Life Technology). Muscle fibres were teased and mounted using Hydromount mounting medium (National Diagnostics). A minimum of 100 NMJs from each sample were randomly selected and captured using confocal laser scanning microscopy. The areas of synapse end plates were measured using ImageJ software.

### Animal procedures

The spontaneous righting reflex was evaluated to estimate muscle strength of mice between PND 1 and PND 20. Mice were placed on their backs, and the time taken to reposition themselves with all four paws on the ground was recorded. The procedure was repeated three times, with at least 5 min recovery period. The maximum recording time is 30 s.

Hind‐limb suspension tests were performed in mice between PND 1 and PND 13, according to the standardized operating procedure recommended by TREAT‐NMD. Mice were suspended by their hind‐limbs from the rim of a 50 mL tube. The posture adopted was scored on a scale of 0 to 4 according to the following criteria: a score of 4 indicates normal hind‐limb separation with tail raised; a score of 3 indicates that weakness is apparent and hind‐limbs are closer but seldom touch each other; a score of 2 indicates that hind‐limbs are close to each other and often touching; a score of 1 indicates that weakness is apparent and the hind‐limbs are almost always in a clasped position with the tail raised; and a score of 0 indicates that constant clasping of the hind‐limbs with the tail lowered or failed to hold onto the tube.

Body weight was measured daily in newborn mice up to 15 days old, followed by measurement every 5 days up to 30 days old and every 10 or 20 days thereafter. Considering the variation in body weights between litters in the neonatal mice with low but rapidly increasing body mass, we decided to use ‘net weight gain’ as the outcome measure to present body weight gain. Briefly, in the same litter, mice were separated into two groups, one group received and another group not received AAV‐MPRO treatment. The ‘net weight gain’ in the litter was the average body weight of mice received AAV‐MPRO minus the average body weight of mice not received AAV‐MPRO. The net weight gains from at least three litters were used for analysis in each experiment.

Hanging wire test was performed in 2‐month‐old mice using the standardized operating procedure of TREAT‐NMD. The mouse was made to grip the wire using the two fore limbs and then released to let it catch the wire with all four limbs. Each mouse was given three trials per session, with 30 s recovery period. The maximum hanging time was recorded by setting the cut‐off time at 120 s, and the average of the three trials was used for statistical analysis.

Treadmill exhaustion tests were performed in adult mice at 30, 45, 60, and 90 days old, according to the standardized operating procedure of TREAT‐NMD. Mice were run on the belt of a motorized treadmill with an orientation of 0° (Omnitech Electronics, Inc.). Mice were acclimatized to a daily running on the treadmill for a week before the test. The treadmill speed was set at 5 m/min for 5 min, followed by 1 m/min increase until exhaustion. The test was terminated when the mouse stopped running at the end of the lane for 20 s despite repeated gentle nudges to make it to reengage on the treadmill. Mice were run to exhaustion, and the time of running and running distance were recorded and calculated, respectively. The cut‐off running time was set at 30 min, which was equal to the maximum running distance of 473 m. Mice were given at least 1 week break between the physical exercise tests and the muscle tissue collection.

### Statistical analysis

The unpaired, two‐tailed Student's *t*‐test or one‐way analysis of variance and post *t*‐test were used to determine statistical significance. Results presented in this study are displayed as mean ± standard error of the mean. Kaplan–Meier survival curves were generated to analyse the survival data, followed by a log‐rank test for statistical significance. Sex‐specific analysis was performed in the body weight gain study. GraphPad Prism 5.0 software was used for statistical analysis and graph design.

## Results

### Adeno‐associated virus‐mediated expression of myostatin propeptide shows modest effect in the severe spinal muscular atrophy mice

Previous studies have suggested that suppression of the myostatin pathway has only limited therapeutic benefit in the severe SMNΔ7 mice.^31^, ^32^, ^33^ To determine the baseline effect of AAV‐MPRO in SMA mice, we injected AAV‐MPRO (2.5 × 10^10^ vp) subcutaneously in the severe Taiwanese SMA mice at PND 0. Body weight, righting reflex, and survival were monitored daily.

**Figure 1:**
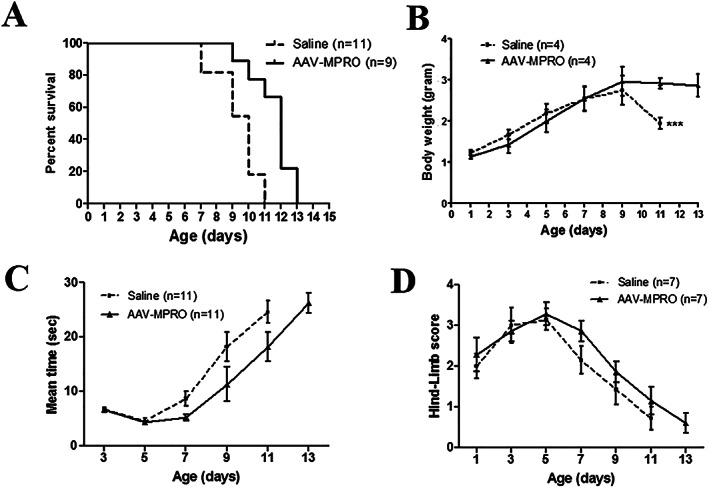
AAV‐MPRO gave only moderate effects on survival, body weight, and motor functions in the severe SMA mice. Severe SMA mice received either saline or a single dose of AAV‐MPRO (2.5 × 10^10^ vp) by subcutaneous injection at PND 0. (A) Kaplan–Meier survival curves of saline‐treated (*n* = 11) and AAV‐MPRO‐treated (*n* = 9) SMA mice (log‐rank *P* = 0.0014). (B) Body weight (g) of saline‐treated and AAV‐MPRO‐treated severe SMA mice (*n* = 4 per group, ^***^ *P* < 0.001, Student's *t*‐test). (C) The ability of righting reflex in SMA mice received saline or AAV‐MPRO treatment (*n* = 11 per group). (D) The recorded hind‐limb suspension score in saline and AAV‐MPRO‐treated SMA mice (*n* = 7 per group).

No significant difference was observed in body weights between saline‐treated and AAV‐MPRO‐treated SMA mice before PND 9. The difference in body weight became significant only after PND 10, when the saline‐treated SMA mice suffered dramatic loss of body weight at the end stage (*Figure* 1A and 1B). The lifespan of SMA mice was modestly increased by AAV‐MPRO, with average survival increased by 2 days (from 10 days in saline‐treated mice to 12 days in AAV‐MPRO‐treated mice) (*Figure* 1A).

To examine the effect of AAV‐MPRO on motor function, we measured righting reflex and hind‐limb suspension in neonatal mice. AAV‐MPRO‐treated SMA mice did not show any improvement in the total righting time or hind‐limb suspension scores, compared with the untreated SMA mice (*Figure* 1C and 1D). Therefore, similar to previous reports,^32^, ^33^ we show that blocking the myostatin pathway using its propeptide resulted in a very modest improvement in mice with severe phenotypes.

### PMO25 increased full‐length SMN2 and myostatin expression

**Figure 2:**
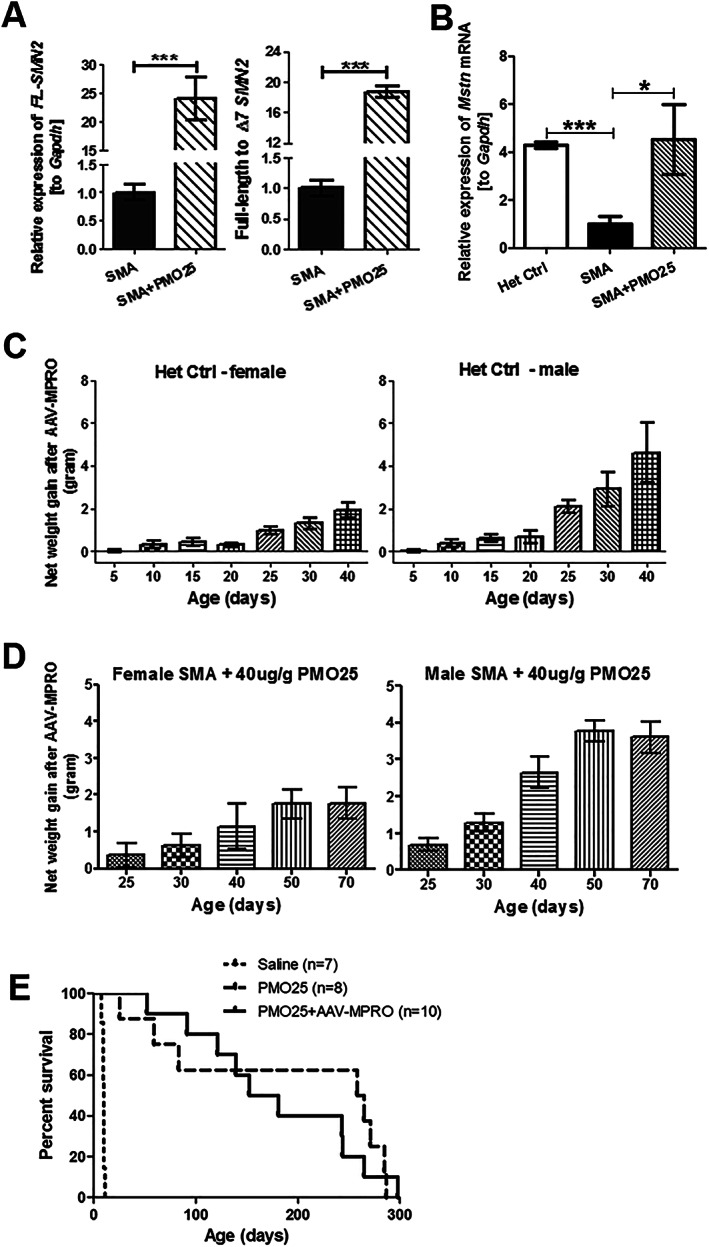
The expression of *Mstn* mRNA in skeletal muscle from SMA mice and the effects of myostatin inhibition on body weight and survival of severe SMA mice receiving 40 μg/g PMO25 treatment. (A) The relative expression of full‐length *SMN2* (FL‐SMN2) transcripts and the ratio of full‐length to Δ7 *SMN2* transcripts in TA muscles from 10‐day‐old untreated SMA mice and SMA mice treated with 40 μg/g PMO25 were measured by real‐time PCR. (*n* = 5 per group, ^***^ *P* < 0.001, Student's *t*‐test). (B) The relative *Mstn* mRNA expression in TA muscles from 10‐day‐old mice was quantified by real‐time PCR. Significant difference was detected between SMA mice and their unaffected littermate controls (Het Ctrl) (*n* = 5 per group, ^*^ *P* < 0.05, ^**^ *P* < 0.01, one‐way analysis of variance and post *t*‐test). Data are represented as mean ± standard error of the mean. (C) The net weight gain (g) in the unaffected heterozygous control (het control) mice. The net weight gain was calculated by deducting the average body weight of the mice without AAV‐MPRO treatment from mice receiving AAV‐MPRO treatment, from the same litter (*n* = 3 litters per group). (D) The net weight gain in SMA mice treated with 40 μg/g PMO25 and AAV‐MPRO, in female and male SMA mice (*n* = 3 litters per group). (E) Kaplan–Meier survival curves of severe SMA mice received saline (*n* = 7), 40 μg/g PMO25 (*n* = 8), and 40 μg/g PMO25 + AAV‐MPRO (*n* = 10). The average survivals were 10, 261, and 166 days, respectively. Log‐rank *P* < 0.0001 between saline control and treated groups; *P* = 0.6292 between PMO25 + AAV‐MPRO and PMO25.

Lower level of circulating myostatin was recently reported in serum from untreated SMA patients and individuals with other dystrophic conditions.^25^ This may explain for the unsatisfactory clinical efficacy of anti‐myostatin approaches in a number of clinical trials for muscular dystrophies. To determine the baseline levels of myostatin in skeletal muscle in SMA mice and its response to SMN‐restoring AON treatment, we performed real‐time PCR to measure the full‐length *SMN2* transcripts, the ratio of full‐length to Δ7 *SMN2* transcripts, and the *Mstn* mRNA expression in mouse skeletal muscles. The effect of PMO25 on augmenting SMN2 exon 7 splicing in skeletal muscle was validated by real‐time PCR in TA muscles collected at PND 10, as previously described.^6^ There was a significant increase of full‐length *SMN2* transcripts and the ratio of full‐length to Δ7 *SMN2* transcripts in PMO25‐treated SMA mice compared with untreated mice (*Figure* 2A). Significant reduction in *Mstn* mRNA expression in TA muscle of 10‐day‐old SMA mice was detected, equal to only 20% of that in the unaffected littermate controls. Interestingly, after a single therapeutic dose (40 μg/g) of PMO25 at PND 0, the *Mstn* mRNA in PMO25‐treated SMA mice was increased to near normal level at PND 10 (*Figure* 2B).

### Myostatin inhibition increases the body weight of therapeutic dose (40 μg/g) antisense oligonucleotide‐treated spinal muscular atrophy mice

We previously reported that a single injection of PMO25 at 40 μg/g increases the survival of SMA mice from 10 days to over 200 days.^6^ However, the treated mice still had significantly smaller muscle fibres than unaffected littermates. To explore whether myostatin inhibition may improve this partially rescued muscle phenotype, we treated SMA mice with PMO25 at 40 μg/g and AAV‐MPRO by a single subcutaneous injection at PND 0. We then evaluated the effects on body weight, muscle mass, survival, and physical performance. Due to the limited effects of AAV‐MPRO therapy on survival of treated SMA mice (to only 12 days), AAV‐MPRO treatment alone and untreated SMA (with a survival time of 10 days) were not included in subsequent experiments. We only compared the combination treatment of PMO25 and AAV‐MPRO with PMO25 treatment alone.

In the unaffected heterozygous control mice, with genotype of (*SMN2*)_2_^+/−^; *Smn* ^+/−^, which were treated with AAV‐MPRO, there was a significant net weight gain from Day 25 onwards. The mean net weight gain in female heterozygous control mice was approximately 0.93 g at Day 25 and 1.9 g at Day 40, an 8% and 9% increase, respectively. In males, the net weight gain was greater—2.1 g at Day 25 and 4.6 g at Day 40, a 12% and 20% increase, respectively (*Figure* 2C).

In SMA mice receiving the combined treatment (PMO25 + AAV‐MPRO), there was a sustaining net weight gain in both male and female mice from Day 25 onwards and reached peak level at Day 50. In female SMA mice, the average net weight gain from Day 50 onwards was over 1.5 g higher than mice receiving PMO25 only, a 9% increase. In males, the average net weight gain after myostatin inhibition was even more significant from Day 50, up to 4 g higher in mice receiving the combinatorial therapy than mice receiving PMO25 only, a 21% increase (*Figure* 2D).

The average survival in SMA mice treated by a single 40 μg/g PMO25 subcutaneous injection was 261 days. There was no additional survival benefit of myostatin inhibition in SMA mice treated with 40 μg/g PMO25. The average survival in SMA mice receiving the combined treatment was slightly lower, down to 166 days. Statistically, there was no difference in survivals between these two groups (*Figure* 2E).

### Myostatin inhibition improves muscle phenotypes and the physical performance in therapeutic dose (40 μg/g) antisense oligonucleotide‐treated spinal muscular atrophy mice

**Figure 3:**
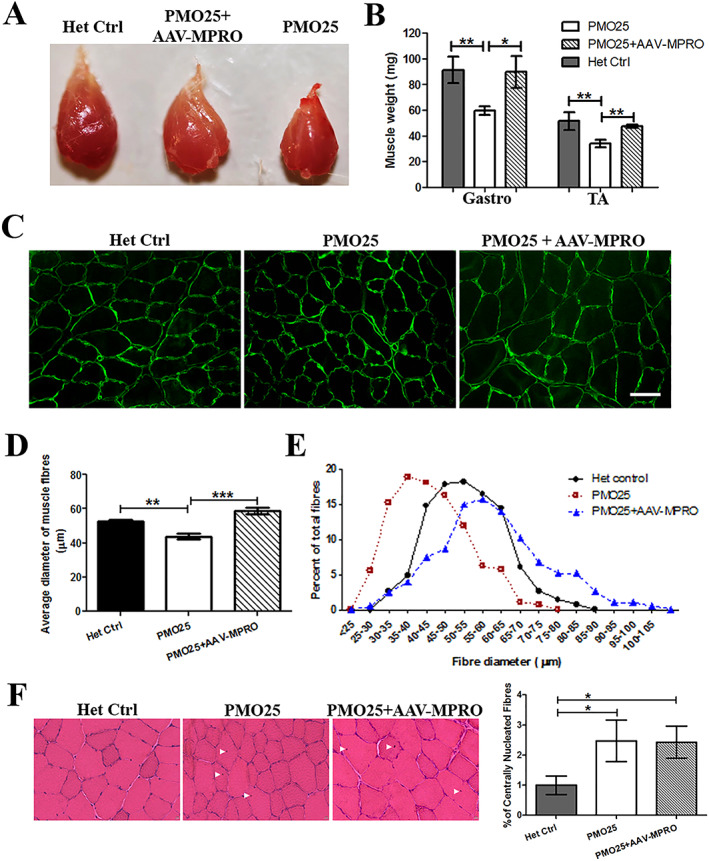
Myostatin inhibition increased muscle mass and fibre size in 40 μg/g PMO25‐treated SMA mice. (A) Representative images of TA muscles dissected from 3‐month‐old female SMA mice receiving different treatments. (B) The addition of AAV‐MPRO increased the weight of TA (Het Ctrl: 51.67 ± 6.89 mg; PMO25 only: 34 ± 3.05 mg; PMO25 + AAV‐MPRO: 47.33 ± 1.2 mg; *n* = 3 mice per group) and gastrocnemius muscle (Het Ctrl: 91.33 ± 10.17 mg; PMO25 only: 59.75 ± 3.42 mg; PMO25 + AAV‐MPRO: 89.75 ± 12.43 mg; *n* = 4 mice per group) in SMA mice receiving 40 μg/g PMO25 treatment. Student's *t*‐test. (C) Representative images of TA muscle fibres stained by laminin (green) for fibre size measurement from mice of different groups. Scale bar = 100 μm. (D) Mean diameters of myofibres from 3‐month‐old SMA mice receiving different treatments (PMO25 + AAV‐MPRO: 58.83 ± 2.074 μm; PMO25 only: 43.83 ± 1.467 μm; het control: 52.41 ± 1.068 μm; *n* = 4 mice per group). Over 200 muscle fibres were assessed in each of three mice. One‐way analysis of variance and post *t*‐test. (E) The percentage of different myofibre diameters from SMA mice receiving different treatments. There was a clear shift from small to large diameter fibres in per cent of total fibres in SMA mice receiving the combined PMO25 + AAV‐MPRO treatment, compared with mice receiving 40 μg/g PMO25 alone. (F) Representative images of haematoxylin and eosin staining of TA muscles and the quantification of centrally nucleated fibres in mice from different groups. The centrally located nuclei were indicated by arrowheads. ^*^ *P* < 0.05, ^**^ *P* < 0.01, ^***^ *P* < 0.001.

We isolated TA and gastrocnemius (gastro) muscles from 3‐month‐old female SMA mice that had received either PMO25 only (40 μg/g) or PMO25 (40 μg/g) + AAV‐MPRO (*Figure* 3A). In agreement with previous studies, AAV‐MPRO significantly increased the weight of TA and gastrocnemius muscles, by 38% and 50%, respectively (*Figure* 3B).

We then measured the size of individual muscle fibres. Laminin staining was used to define the muscle fibres boundaries for fibre diameter measurement. The addition of AAV‐MPRO increased the size of muscle fibres by 35%, compared with SMA mice that received PMO25 only (*Figure* 3C and 3D). The spectrum of the myofibre diameter in SMA mice receiving combined treatment was shifted to larger diameter fibres, compared with mice receiving PMO25 only (*Figure* 3E).

Haematoxylin and eosin staining was performed on transverse sections of TA muscles, for evaluation of the muscle histopathology. There were increased centrally located nuclei (evidence of past myofibre regeneration) in SMA mice treated with PMO (2.5%) or PMO + AAV‐MPRO (2.4%) compared with unaffected heterozygous controls (1%) (*Figure* 3F). There was no difference in the percentage of centrally nucleated fibres between PMO and PMO + AAV‐MPRO groups, indicating that myostatin inhibition has no effect on skeletal muscle regeneration/degeneration in PMO25‐treated SMA mice.

**Figure 4:**
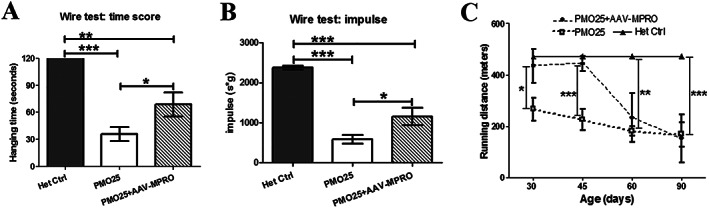
Myostatin inhibition increased the physical performance in 40 μg/g PMO25‐treated SMA mice. (A) The hanging wire grip tests were performed to assess muscle strength in 2‐month‐old SMA mice receiving 40 μg/g PMO25, with (68.71 ± 13.41 s; *n* = 7) or without (35.83 ± 7.67 s; *n* = 6; *P* = 0.0336) AAV‐MPRO treatment. All the unaffected heterozygous control mice (*n* = 5) reached the 120 s cut‐off time. (B) Holding impulses were calculated where the impact of body weight was considered. The holding impulse was 585 ± 106 in PMO25 only group (*n* = 6), 1151 ± 219 in AAV‐MPRO + PMO25 group (*n* = 7) and 2376 ± 46.63 in the heterozygous control group (*n* = 5). (C) Treadmill exercise tests were performed at the ages of 30, 45, 60, and 90 days after mice receiving different treatments at birth. The running distances (m) were calculated in different groups. All the unaffected heterozygous control mice reached the 473 m cut‐off distance. *N* = 3–5 mice per group. ^*^ *P* < 0.05, ^**^ *P* < 0.01, ^***^ *P* < 0.001.

It has been reported that myostatin inhibition increases the absolute force production in skeletal muscle of the milder SMA^C/C^ mouse model.^34^ We wanted to examine whether the combined treatment could improve the therapeutic effect of SMN‐restoring AON treatment at functional level. To determine the physical performance on different treatments, mice were tested by hanging wire grip test and treadmill exercise. The hanging wire grip was tested in 2‐month‐old SMA mice that received 40 μg/g PMO25 at PND 0, with or without AAV‐MPRO treatment. While all the unaffected heterozygous control mice reached the 120 s cut‐off time, SMA mice on 40 μg/g PMO25 alone were only able to maintain their grip for 35 s. The addition of AAV‐MPRO increased the latency‐to‐fall approximately two‐fold to 68 s (*Figure* 4A).

Considering the possible impact of body weight on the hanging wire test, we used the Holding Impulse (s*g) = Body mass (g) × Hanging time (s) as an outcome measure (http://www.treat‐nmd.eu). This reflects the tension (impulse) that the animal develops in order to maintain itself on the wire, against gravity for the longest period. There was a significant difference in impulse between mice treated with PMO25 only and those treated with AAV‐MPRO + PMO25 (*Figure* 4B). Results from both assessments indicate that the addition of AAV‐MPRO increases muscle strength in PMO25‐treated SMA mice.

Treadmill exercise tests were performed to evaluate the efficacy of AAV‐MPRO and PMO25 on physical performance. Experiments were performed at the ages of 30, 45, 60, and 90 days, respectively. All the unaffected heterozygous control mice reached the 473 m cut‐off distance. SMA mice treated with PMO25 only showed a constant tolerance to exercise, with a mean running distance around 200 m (*Figure* 4C). There was a significant increase in running distance in mice receiving the combined treatments at both 30 and 45 days of age, when compared with the PMO25 only littermates. However, the running distance in PMO25 + AAV‐MPRO group declined sharply from 45 to 60 days and reached a similar level to the PMO25 only mice at around 60–90 days old (*Figure* 4C).

### Myostatin inhibition improves the phenotype of low‐dose (10 μg/g) antisense oligonucleotide‐treated spinal muscular atrophy mice

**Figure 5:**
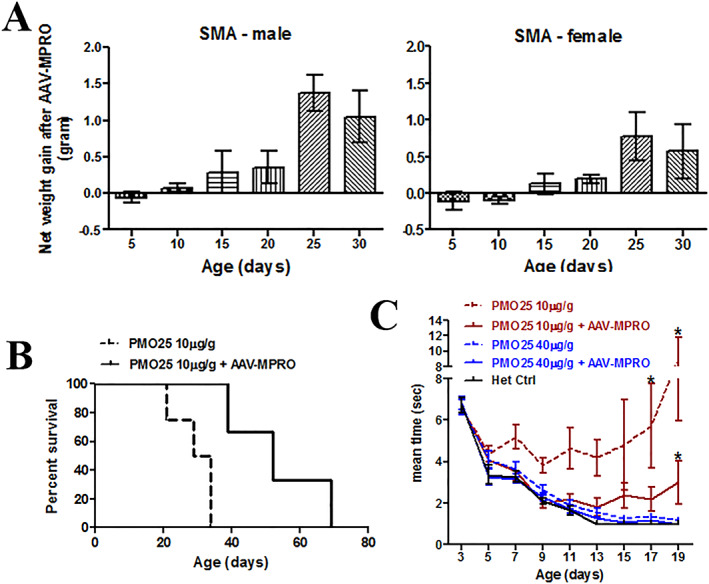
AAV‐MPRO improved the general phenotypes in low‐dose PMO25‐treated (10 μg/g) SMA mice. (A) The net body weight gain (g) in SMA mice treated with low‐dose PMO25 at 10 μg/g and AAV‐MPRO. The average body weight of SMA mice receiving both PMO25 and AAV‐MPRO treatment deducted the average body weight of SMA mice received PMO25 only, from the same litter (*n* = 3 litters per group). The net weight gains in female and male mice were calculated separately. (B) Kaplan–Meier survival curves of severe SMA mice received 10 μg/g PMO25 (*n* = 4) and 10 μg/g PMO25 + AAV‐MPRO (*n* = 3) (log‐rank *P* = 0.021). (C) The abilities of righting reflex in SMA mice receiving 10 μg/g PMO25, 10 μg/g PMO25 + AAV‐MPRO, 40 μg/g PMO25, 10 μg/g PMO25 + AAV‐MPRO and the het control (*n* = 11 per group). Significant differences were detected between SMA mice receiving 10 μg/g PMO25 and SMA mice receiving 10 μg/g PMO25 + AAV‐MPRO on Days 17 and 19, as well as between mice receiving 10 μg/g PMO25 + AAV‐MPRO and mice receiving 40 μg/g PMO25 + AAV‐MPRO on Day 19. ^*^ *P* < 0.05, Student's *t*‐test.

We next sought to obtain more information on the potential effect of myostatin inhibition in the less severe and more chronic forms of SMA, that is, Types II and III SMA, in whom combinatorial therapies could be envisioned. We designed a series of experiments in mice with the ‘intermediate’ phenotypes, a model previously generated using low‐dose PMO25 in the severe SMA mice.^29^ SMA mice treated with a single dose of 10 μg/g PMO25 at PND 0 had a moderate increase in lifespan and a modest but significant improvement in motor function and neuromuscular pathology, in comparison with the untreated severe SMA mice.^29^ In this study, we administered AAV‐MPRO together with 10 μg/g PMO25 (AAV‐MPRO + 10 μg/g PMO25) at PND 0 and compared with mice that received 10 μg/g PMO25 only. There was a significant body weight gain from Day 25 in SMA mice that received the combined treatment. The net weight gain in AAV‐MPRO + 10 μg/g PMO25‐treated mice, by deducting the mean body weight of SMA mice receiving only 10 μg/g PMO25 in the same litter, was more dramatic in males than females. In SMA mice treated with 10 μg/g PMO25, the mean net weight gain at Day 25 is 1.37 g in males (a 9% increase) and 0.78 g in females (a 7% increase) (*Figure* 5A).

The combined treatment significantly increased the survival of SMA mice compared with mice receiving 10 μg/g PMO25 only, as represented by average survival of 52 days vs. 31 days (a 40% increase) (*Figure* 5B). We also assessed the functional consequences of the combined treatment, compared with mice receiving only 10 μg/g PMO25. SMA mice that received combined treatment had a much shorter righting reflex time than those receiving 10 μg/g PMO25 only, after 17 days of age, although this was still inferior to the improvement achieved in SMA mice treated with 40 μg/g PMO25 and the heterozygous control mice (*Figure* 5C).

### Adeno‐associated virus‐mediated expression of myostatin propeptide improves neuromuscular junction maturation and preserves the proprioceptive synapses in low‐dose (10 μg/g) antisense oligonucleotide‐treated spinal muscular atrophy mice

**Figure 6:**
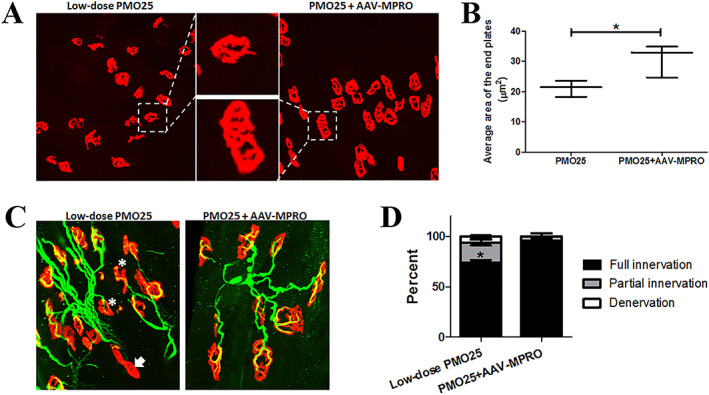
AAV‐MPRO improved NMJ maturation and innervation in low‐dose PMO25‐treated (10 μg/g) SMA mice. (A) Representative images of motor end plates, stained by α‐bungarotoxin (red), in TA muscles of 20‐day‐old SMA mice from different groups. SMA mice receiving the combinatorial treatment (PMO25 + AAV‐MPRO) showed mature morphology of the end plates, with increased length and branching of the post‐synaptic membrane and enlargement of the post‐synaptic area. (B) The areas of end plates (μm^2^) were quantified by ImageJ software. Over 200 end plates were assessed in each mouse. The average end plate size (μm^2^) was 30.82 ± 3.12 in PMO25 + AAV‐MPRO group and 21.12 ± 1.55 in mice receiving PMO25 only (*n* = 3 per group; *P* = 0.0248). (C) Representative images of NMJs in TA muscles from 20‐day‐old mice from different groups. The end plates were stained with α‐bungarotoxin (red), and the nerves were stained with anti‐neurofilament and anti‐synaptophysin (green). Asterisk indicates the partial innervation, and arrow indicates the denervation. (D) Histograms showing the quantification of fully innervated, partially innervated, and fully denervated end plates in TA muscles at PND 20 in different groups. ^*^ *P* < 0.05. Student's *t*‐test.

The effect of myostatin inhibition on the survival in low‐dose PMO25‐treated SMA prompted us to study in more detail the effect of this approach on different anatomical locations. To assess the effect of myostatin inhibition on the development of NMJs in the low‐dose PMO25‐treated SMA mice, we examined the morphology of end plates in TA muscle collected at PND 20. The ‘plaque‐to‐pretzel’ transition of the end plate is an indicator of post‐synaptic maturation.^35^ The end plates in SMA mice receiving the combined treatment had a more mature morphology, characterized by increased length and branching of the post‐synaptic membrane with enlargement of the post‐synaptic area (pretzel‐like shape). There was a significant increase in end plate size in SMA mice receiving combined treatment compared with mice receiving PMO25 only (*Figure* 6A and 6B). In addition, myostatin inhibition significantly increased the percentage of fully innervated NMJs in low‐dose PMO25‐treated SMA mice (*Figure* 6C and 6D).

**Figure 7:**
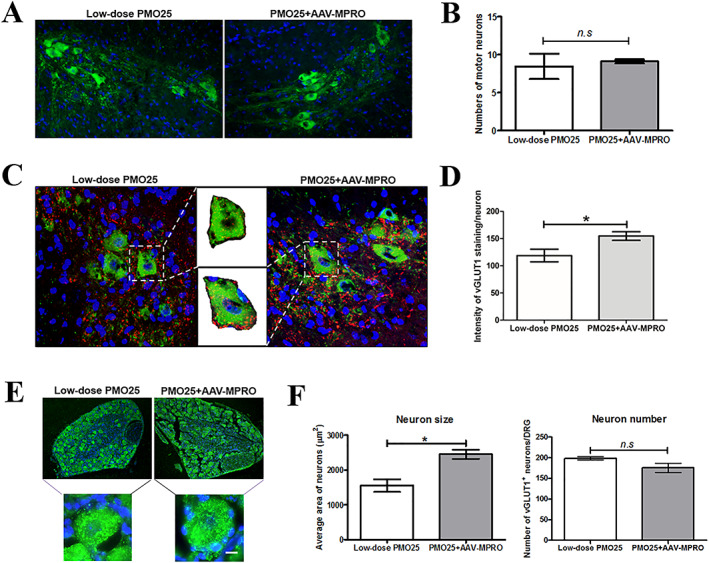
AAV‐MPRO improved the preservation of proprioceptive synapses and the sizes of sensory neurons in low‐dose PMO25‐treated (10 μg/g) SMA mice. (A) Representative images of choline acetyltransferase‐positive motor neurons in lumbar spinal cord in mice at PND 20. (B) Quantity of motor neuron counts in SMA mice receiving PMO25 + AAV‐MPRO treatment or PMO25 alone (n = 3 mice per group). (C) Representative micrographs of confocal images of choline acetyltransferase‐positive motor neurons (green) and vGLUT1‐positive synapses (red) in lumbar spinal cord from 20‐day‐old SMA mice receiving different treatments. Representative enlarged images of single motor neuron with area of interest (AOI), which was used for vGLUT1 intensity quantification were inserted. (D) Quantification of vGLUT1^+^ synapses per choline acetyltransferase‐positive motor neurons in the ventral horn of the lumbar spinal cord sections from mice receiving different treatments. (E) Representative images of vGLUT1^+^ sensory neurons in DRG from 20‐day‐old mice receiving different treatments. Representative enlarged images of a single sensory neuron in each group were inserted. (F) Quantification of the size of vGLUT1^+^ sensory neurons in DRGs from SMA mice receiving different treatments (*n* = 3 per group). ^*^ *P* < 0.05. Student's *t*‐test.

To explore if myostatin inhibition has any additive effects on the survival of motor neurons, which may contribute to the prolonged lifespan in low‐dose PMO25‐treated SMA mice, we counted the number of motor neurons in the lumbar spinal cords in PND 20 mice. However, there was no difference between the two groups (*Figure* 7A and 7B).

Vesicular glutamate transporter 1(vGLUT1) is a marker of synaptic vesicles in primary afferent terminals and has been used to assess the integrity of the synapse of proprioceptive sensory neurons in SMA mice.^36^ Reduction of proprioceptive synapses on the lumbar motor neurons has been reported in SMA mice.^37^ Interestingly, here, we detected a significant increase in vGLUT1^+^ synapses in the ventral horn area around the choline acetyltransferase‐positive motor neurons in mice receiving the combined treatment than those receiving low‐dose PMO25 only (*Figure* 7C and 7D).

To further understand the effect of myostatin inhibition on sensory neurons, we dissected DRG of the lumbar segment and immunostained the sensory neurons with anti‐vGLUT1 antibody. We found that the size of vGLUT1^+^ sensory neurons in DRGs was significantly increased in SMA mice receiving both PMO25 and AAV‐MPRO compared with low‐dose PMO25 only. There was no significant difference in the number of sensory neurons between these two groups (*Figure* 7E and 7F).

## Discussion

The characteristic muscular atrophy in SMA is largely secondary to the denervation resulted from spinal motor neuron death. Nevertheless, there is evidence that, especially following severe depletion of SMN, intrinsic abnormalities in skeletal muscle cells also contribute to disease progression. Myoblasts expressing low levels of SMN protein have reduced proliferation, fusion, and myotube formation, suggesting a cell autonomous defect independent of innervation.^38^, ^39^ Prenatal delay in muscle growth and maturation has been reported in severe SMA mice, as indicated by drastically smaller myotubes when the muscle was cultured *in vitro*.^40^ This is at least partially attributed to the impairment in the myogenic programme.^41^ It is plausible that muscle‐enhancing approach by improving skeletal muscle growth and maturation via the blockage of myostatin pathways could be beneficial to SMA.

However, inhibition of the myostatin pathways gives only little or no effect in the severe SMA transgenic mice.^31^, ^32^, ^33^ In agreement with these observations, we showed in this study that systemic administration of AAV‐MPRO in neonatal severe SMA mice had only a very modest effect on survival (*Figure* 1). This is likely due to the extremely low level of endogenous SMN protein and the very short lifespan of the severe SMA mice. As discussed further in the succeeding text, the reduced levels of myostatin in skeletal muscle in SMA mice could also contribute to this outcome.

We have assessed the effect of systemic inhibition of myostatin by AAV‐MPRO in the severe SMA mice receiving 40 μg/g PMO25 treatment. As expected, myostatin inhibition increased the net weight gain, skeletal muscle mass, and fibre size. The increased muscle strength in SMA mice receiving the combinatorial treatment in our study (*Figure* 4) is consistent with the recent report on increased torque force observed in SMA mice receiving pharmacological restoration of SMN by small molecule and myostatin monoclonal antibody.^26^ Surprisingly, in our functional study on physical endurance, the beneficial effect was maximum in the first 2 months of life but declined in treated mice older than 2 months of age (*Figure* 4). This may be due to the effect of myostatin inhibition on redox homeostasis and mitochondrial function in skeletal muscle. The lack of myostatin in Mstn‐knockout mice decreases the coupling of intermyofibrillar mitochondria while significantly increasing the basal oxygen consumption.^42^ Indeed, it has been reported previously that either Mstn‐knockout mice or adult wild type mice receiving myostatin blockade by AAV‐MPRO had decreased endurance exercise capacity, with decreased mitochondrial respiration, increased enolase activity, and exercise‐induced lactic acidosis, suggesting the shift of muscle from aerobic towards anaerobic energy metabolism.^43^ The constitutive complete blockage of myostatin signalling diminishes exercise capacity, while the overgrowth of muscle mass increases oxygen consumption and the energy cost of running. Therefore, as muscles become larger and stronger, they fatigue more rapidly in myostatin deficiency.^43^ It is also noted that in the SMA mice receiving 40 μg/g PMO25 treatment at PND 0, myostatin inhibition did not show additional benefit on survival (*Figure* 2E). These results suggest that myostatin inhibition may confer limited functional advantage in AON‐treated SMA mice at later stage of disease, likely ascribed to the increased fatigability.

A recent study on myostatin pathways in neuromuscular diseases has demonstrated the extremely low baseline level of myostatin in patients with muscle wasting or atrophic diseases and in particular in DMD and SMA.^25^ In keeping with the data reported in SMA patients, we confirm in this study that the *Mstn* mRNA transcripts was downregulated by ~80% in skeletal muscles in the severe SMA mice compared with that of the unaffected littermate controls. An important observation of our study however is that *Mstn* mRNA expression reverses back to near normal level after effective AON treatment (*Figure* 2B). Our result confirms that one of the reasons for the little or no effect of myostatin inhibition in the severe SMA mice is likely due to the extremely low levels of endogenous myostatin, hence very limited target to engage. These data also support the idea that myostatin could represent a biomarker of response to therapeutic intervention following SMN‐enhancing strategies.^25^ Furthermore, these data provide the rationale for the development of the combinatorial therapy for SMA, as the AON treatment increases the levels of myostatin inhibition therapeutic targets, enhancing the target engagement of this combined therapy.

In this study, we also explored the potential clinical benefit of myostatin inhibition on mice treated with lower doses of AON, aimed at mimicking the situation of patients with the chronic forms of SMA II and III and those who may be under insufficient SMN‐restoring drug treatment. Encouraging results have recently presented that myostatin inhibition exerts therapeutic effect in SMA mice with less severe phenotypes: in particular, inhibition of myostatin using intramuscular injection of AAV1‐follistatin or monoclonal antibody ameliorates muscle atrophy in the Δ7 SMA mice dosed with low, sub‐optimal doses of an *SMN2* splicing modifier small molecule.^26^, ^27^ Intraperitoneal administration of AAV mediated a soluble form of the ActRIIB extracellular domain, by blocking the activin receptor, and also elicited great improvement in muscle mass and force in the mild SMA^C/C^ mouse model.^34^ These studies indicate that myostatin pathway inhibition could be considered as a potential therapy for patients with milder forms of SMA. In this study, we showed that myostatin inhibition can notably improve the survival, in addition to the effect on body weight gain and righting reflex improvement, in low‐dose PMO25 treated severe SMA mice (*Figure* 5). This promising result presents further evidence that myostatin inhibition may benefit survival, although differences in the efficacy in different age ranges and gender could be expected. To explore the possible underlying mechanisms, we examined the phenotypes of the NMJs and neurons in DRG and the spinal cord.

**Figure 8:**
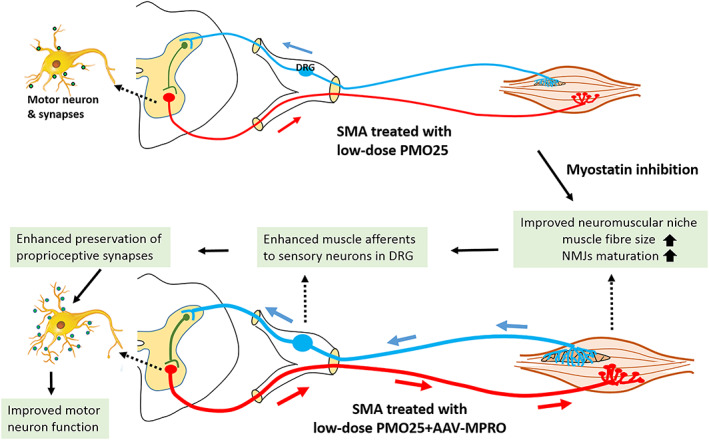
Schematic illustration of the neural circuit between central nervous neurons and the muscle motor units. It is hypothesized that the enhanced muscle niche by myostatin inhibition may send stronger afferents (e.g. via muscle spindle) to sensory neurons in DRG, improve the preservation of proprioceptive synapses on motor neurons in spinal cord, and enhance the function of motor neurons.

Recent studies in SMA transgenic mice indicate the involvement of spinal and neuromuscular circuitry in the pathogenesis of the disease.^36^, ^44^ The functional impairment of sensory–motor connectivity, reflected by the reduced proprioceptive reflexes and the decreased number and function of afferent synapses on motor neurons,^36^ may occur prior to the loss of mobility in patients and the loss of motor neurons. Dysfunction of proprioceptive synapses, characterized by reduced glutamate release, impairs motor neuron function in SMA.^45^ Inhibitory effect of myostatin on synaptic function and composition at the larval NMJ, and synaptogenesis in isolated rat cortical neuron cultures have been reported previously.^46^ Here, we found that the mice receiving combined treatment have more mature NMJs than those who received AON only. In addition, although no effect on the number of spinal motor neurons was demonstrated, there was a significant increase of vGLUT1^+^ proprioceptive afferent synapses in lumbar spinal cord in the ‘intermediate’ mice receiving AAV‐MPRO (*Figure* 7C and 7D). Consistent with this result, we find that sensory neurons were larger in DRG in mice receiving the additional AAV‐MPRO treatment (*Figure* 7E and 7F). Based on all these data, we hypothesize that the improved muscle health and maturation induced by myostatin inhibition may enhance sensory afferences (via muscle spindle or perisynaptic Schwann cells at NMJs) to sensory neurons in DRG, improve the preservation of proprioceptive synapses on motor neurons in spinal cord, enhance the function of motor neurons, and eventually increase survival (*Figure* 8). This hypothesis is also supported by the fact that axonal hypertrophy of nerves present in muscles and muscle spindle hyperplasia was observed in the germline myostatin null mice.^47^

It is noteworthy that, in addition to skeletal muscle, *Mstn* transcript and receptors are also expressed in brain and peripheral nerves.^48^ An inhibitory effect of a myostatin‐like protein, growth differentiation factor 11, was reported on neuronal colony formation *in vitro,* 49 synaptic function and composition at the larval NMJs, and synaptogenesis in isolated rat cortical neuron cultures.^46^ Growth differentiation factor 11 and myostatin are closely related members of the TGF‐β superfamily and share high sequence similarity.^50^ It is unclear whether the myostatin blockage pathway we performed in newborn SMA mice had any direct effect, or indirect effect via other myostatin‐like proteins, on synapse formation and motor neuron function. Further experiments designed to determine the effect of myostatin inhibition on motor neurons will help to answer this question.

In this study, we showed for the first time the benefit of myostatin inhibition to motor function and its potential effect on the neuromuscular pathologies in SMA. This effect occurs not only in skeletal muscle but also in the neural circuits with potential benefit on motor neurons. Our results shed further light on the complex relationship between skeletal muscle, NMJ, and motor neurons, which should be considered as a unit when developing novel therapies. Our study provides further evidence for developing a combinatorial therapy by using myostatin inhibitors and Spinraza or other SMN‐restoring drugs for SMA.

## Author Contributions

H.Z., A.M., G.D., and F.M. conceptualized the study. H.Z., G.D., and F.M. provided funding. H.Z., J.M., F.C., A.M., L.S., N.L.‐N., J.D., V.M., J.E.M., and P.S. performed and analysed the experiments. A.M., S.J., N.L.‐N., and G.D. produced and provided AAV‐MPRO viral particles. H.Z., J.M., A.M., G.D., J.E.M., P.G., and F.M. wrote and discussed the manuscript, which was approved by all authors. H.Z. and F.M. supervised the project.

## Conflicts of Interest

F.M. has served on scientific advisory boards for Sarepta, Pfizer, Roche Biogen, and Avexis, receives research support from Biogen, and has received funding for trials from Sarepta, Avexis, Biogen, PTC, Wave, Roche, and Sarepta Therapeutics. G.D. has served on scientific advisory boards for Benitec, Freeline, and Genethon and has received commercial support for research from Sarepta, Pfizer, Benitec, and SynPromics. The remaining authors declare no conflicts of interest in this study. V.M. and J.D. are inventors of the patent PCT/GB2018/050619 filled by UCL Business.

## References

[1] Lefebvre S , Burglen L , Reboullet S , Clermont O , Burlet P , Viollet L , et al. Identification and characterization of a spinal muscular atrophy‐determining gene. Cell 1995;80:155–165. 7813012 10.1016/0092-8674(95)90460-3

[2] Lorson CL , Hahnen E , Androphy EJ , Wirth B . A single nucleotide in the SMN gene regulates splicing and is responsible for spinal muscular atrophy. Proceedings of the National Academy of Sciences of the United States of America 1999;96:6307–6311. 10339583 10.1073/pnas.96.11.6307 PMC26877

[3] Monani UR , Lorson CL , Parsons DW , Prior TW , Androphy EJ , Burghes AH , et al. A single nucleotide difference that alters splicing patterns distinguishes the SMA gene SMN1 from the copy gene SMN2. Human Molecular Genetics 1999;8:1177–1183. 10369862 10.1093/hmg/8.7.1177

[4] Hua Y , Sahashi K , Rigo F , Hung G , Horev G , Bennett CF , et al. Peripheral SMN restoration is essential for long‐term rescue of a severe spinal muscular atrophy mouse model. Nature 2011;478:123–126. 21979052 10.1038/nature10485 PMC3191865

[5] Porensky PN , Mitrpant C , McGovern VL , Bevan AK , Foust KD , Kaspar BK , et al. A single administration of morpholino antisense oligomer rescues spinal muscular atrophy in mouse. Human Molecular Genetics 2012;21:1625–1638. 22186025 10.1093/hmg/ddr600 PMC3298284

[6] Zhou H , Janghra N , Mitrpant C , Dickinson R , Anthony K , Price L , et al. A novel morpholino oligomer targeting ISS‐N1 improves rescue of severe SMA transgenic mice. Human Gene Therapy 2013;24:331–342. 23339722 10.1089/hum.2012.211 PMC3609631

[7] Passini MA , Bu J , Richards AM , Bevan AK , Foust KD , Kaspar BK , et al. Antisense oligonucleotides delivered to the mouse CNS ameliorate symptoms of severe spinal muscular atrophy. Science Translational Medicine 2011;3:72ra18. 10.1126/scitranslmed.3001777 PMC3140425 21368223

[8] Foust KD , Wang X , McGovern VL , Braun L , Bevan AK , Haidet AM , et al. Rescue of the spinal muscular atrophy phenotype in a mouse model by early postnatal delivery of SMN. Nature Biotechnology 2010;28:271–274. 10.1038/nbt.1610 PMC2889698 20190738

[9] Valori CF , Ning K , Wyles M , Mead RJ , Grierson AJ , Shaw PJ , et al. Systemic delivery of scAAV9 expressing SMN prolongs survival in a model of spinal muscular atrophy. Science Translational Medicine 2010;2:35ra42. 10.1126/scitranslmed.3000830 20538619

[10] Passini MA , Bu J , Roskelley EM , Richards AM , Sardi SP , O'Riordan CR , et al. CNS‐targeted gene therapy improves survival and motor function in a mouse model of spinal muscular atrophy. The Journal of Clinical Investigation 2010;120:1253–1264. 20234094 10.1172/JCI41615 PMC2846065

[11] Naryshkin NA , Weetall M , Dakka A , Narasimhan J , Zhao X , Feng Z , et al. Motor neuron disease. SMN2 splicing modifiers improve motor function and longevity in mice with spinal muscular atrophy. Science 2014;345:688–693. 25104390 10.1126/science.1250127

[12] Scoto M , Finkel RS , Mercuri E , Muntoni F . Therapeutic approaches for spinal muscular atrophy (SMA). Gene Therapy 2017;24:514–519. 28561813 10.1038/gt.2017.45

[13] Finkel RS , Mercuri E , Darras BT , Connolly AM , Kuntz NL , Kirschner J , et al. Nusinersen versus sham control in infantile‐onset spinal muscular atrophy. The New England Journal of Medicine 2017;377:1723–1732. 29091570 10.1056/NEJMoa1702752

[14] Mercuri E , Darras BT , Chiriboga CA , Day JW , Campbell C , Connolly AM , et al. Nusinersen versus sham control in later‐onset spinal muscular atrophy. The New England Journal of Medicine 2018;378:625–635. 29443664 10.1056/NEJMoa1710504

[15] McPherron AC , Lawler AM , Lee SJ . Regulation of skeletal muscle mass in mice by a new TGF‐β superfamily member. Nature 1997;387:83–90. 9139826 10.1038/387083a0

[16] Lee SJ , McPherron AC . Regulation of myostatin activity and muscle growth. Proceedings of the National Academy of Sciences of the United States of America 2001;98:9306–9311. 11459935 10.1073/pnas.151270098 PMC55416

[17] Zhu X , Hadhazy M , Wehling M , Tidball JG , McNally EM . Dominant negative myostatin produces hypertrophy without hyperplasia in muscle. FEBS Letters 2000;474:71–75. 10828454 10.1016/s0014-5793(00)01570-2

[18] Hill JJ , Davies MV , Pearson AA , Wang JH , Hewick RM , Wolfman NM , et al. The myostatin propeptide and the follistatin‐related gene are inhibitory binding proteins of myostatin in normal serum. The Journal of Biological Chemistry 2002;277:40735–40741. 12194980 10.1074/jbc.M206379200

[19] Hennebry A , Berry C , Siriett V , O'Callaghan P , Chau L , Watson T , et al. Myostatin regulates fiber‐type composition of skeletal muscle by regulating MEF2 and MyoD gene expression. American Journal of Physiology. Cell Physiology 2009;296:C525–C534. 19129464 10.1152/ajpcell.00259.2007

[20] Matsakas A , Foster K , Otto A , Macharia R , Elashry MI , Feist S , et al. Molecular, cellular and physiological investigation of myostatin propeptide‐mediated muscle growth in adult mice. Neuromuscular Disorders 2009;19:489–499. 19541486 10.1016/j.nmd.2009.06.367

[21] Kang JK , Malerba A , Popplewell L , Foster K , Dickson G . Antisense‐induced myostatin exon skipping leads to muscle hypertrophy in mice following octa‐guanidine morpholino oligomer treatment. Molecular Therapy 2011;19:159–164. 20924365 10.1038/mt.2010.212 PMC3017443

[22] Malerba A , Kang JK , McClorey G , Saleh AF , Popplewell L , Gait MJ , et al. Dual myostatin and dystrophin exon skipping by morpholino nucleic acid oligomers conjugated to a cell‐penetrating peptide is a promising therapeutic strategy for the treatment of Duchenne muscular dystrophy. Molecular Therapy‐‐Nucleic Acids 2012;1:e62. 23250360 10.1038/mtna.2012.54 PMC3528303

[23] Dumonceaux J , Marie S , Beley C , Trollet C , Vignaud A , Ferry A , et al. Combination of myostatin pathway interference and dystrophin rescue enhances tetanic and specific force in dystrophic mdx mice. Molecular Therapy 2010;18:881–887. 20104211 10.1038/mt.2009.322 PMC2890116

[24] Qiao C , Li J , Jiang J , Zhu X , Wang B , Li J , et al. Myostatin propeptide gene delivery by adeno‐associated virus serotype 8 vectors enhances muscle growth and ameliorates dystrophic phenotypes in mdx mice. Human Gene Therapy 2008;19:241–254. 18288893 10.1089/hum.2007.159

[25] Mariot V , Joubert R , Hourde C , Feasson L , Hanna M , Muntoni F , et al. Downregulation of myostatin pathway in neuromuscular diseases may explain challenges of anti‐myostatin therapeutic approaches. Nature Communications 2017;8:1859. 10.1038/s41467-017-01486-4 PMC5709430 29192144

[26] Long KK , O'Shea KM , Khairallah RJ , Howell K , Paushkin S , Chen KS , et al. Specific inhibition of myostatin activation is beneficial in mouse models of SMA therapy. Human Molecular Genetics 2019;28:1076–1089. 30481286 10.1093/hmg/ddy382 PMC6423420

[27] Feng Z , Ling KK , Zhao X , Zhou C , Karp G , Welch EM , et al. Pharmacologically induced mouse model of adult spinal muscular atrophy to evaluate effectiveness of therapeutics after disease onset. Human Molecular Genetics 2016;25:964–975. 26758873 10.1093/hmg/ddv629

[28] Hsieh‐Li HM , Chang JG , Jong YJ , Wu MH , Wang NM , Tsai CH , et al. A mouse model for spinal muscular atrophy. Nature Genetics 2000;24:66–70. 10615130 10.1038/71709

[29] Zhou H , Meng J , Marrosu E , Janghra N , Morgan J , Muntoni F . Repeated low doses of morpholino antisense oligomer: an intermediate mouse model of spinal muscular atrophy to explore the window of therapeutic response. Human Molecular Genetics 2015;24:6265–6277. 26264577 10.1093/hmg/ddv329 PMC4614699

[30] Foster K , Graham IR , Otto A , Foster H , Trollet C , Yaworsky PJ , et al. Adeno‐associated virus‐8‐mediated intravenous transfer of myostatin propeptide leads to systemic functional improvements of slow but not fast muscle. Rejuvenation Research 2009;12:85–94. 19405813 10.1089/rej.2008.0815

[31] Rose FF Jr , Mattis VB , Rindt H , Lorson CL . Delivery of recombinant follistatin lessens disease severity in a mouse model of spinal muscular atrophy. Human Molecular Genetics 2009;18:997–1005. 19074460 10.1093/hmg/ddn426 PMC2649020

[32] Sumner CJ , Wee CD , Warsing LC , Choe DW , Ng AS , Lutz C , et al. Inhibition of myostatin does not ameliorate disease features of severe spinal muscular atrophy mice. Human Molecular Genetics 2009;18:3145–3152. 19477958 10.1093/hmg/ddp253 PMC2733819

[33] Rindt H , Buckley DM , Vale SM , Krogman M , Rose FF , Garcia ML , et al. Transgenic inactivation of murine myostatin does not decrease the severity of disease in a model of Spinal Muscular Atrophy. Neuromuscular Disorders 2012;22:277–285. 22079083 10.1016/j.nmd.2011.10.012

[34] Liu M , Hammers DW , Barton ER , Sweeney HL . Activin receptor type IIB inhibition improves muscle phenotype and function in a mouse model of spinal muscular atrophy. PLoS ONE 2016;11:e0166803. 27870893 10.1371/journal.pone.0166803 PMC5117715

[35] Marques MJ , Conchello JA , Lichtman JW . From plaque to pretzel: fold formation and acetylcholine receptor loss at the developing neuromuscular junction. The Journal of Neuroscience 2000;20:3663–3675. 10804208 10.1523/JNEUROSCI.20-10-03663.2000 PMC6772702

[36] Mentis GZ , Blivis D , Liu W , Drobac E , Crowder ME , Kong L , et al. Early functional impairment of sensory‐motor connectivity in a mouse model of spinal muscular atrophy. Neuron 2011;69:453–467. 21315257 10.1016/j.neuron.2010.12.032 PMC3044334

[37] Ling KK , Lin MY , Zingg B , Feng Z , Ko CP . Synaptic defects in the spinal and neuromuscular circuitry in a mouse model of spinal muscular atrophy. PLoS ONE 2010;5:e15457. 21085654 10.1371/journal.pone.0015457 PMC2978709

[38] Hayhurst M , Wagner AK , Cerletti M , Wagers AJ , Rubin LL . A cell‐autonomous defect in skeletal muscle satellite cells expressing low levels of survival of motor neuron protein. Developmental Biology 2012;368:323–334. 22705478 10.1016/j.ydbio.2012.05.037 PMC3851302

[39] Shafey D , Cote PD , Kothary R . Hypomorphic Smn knockdown C2C12 myoblasts reveal intrinsic defects in myoblast fusion and myotube morphology. Experimental Cell Research 2005;311:49–61. 16219305 10.1016/j.yexcr.2005.08.019

[40] Martinez‐Hernandez R , Soler‐Botija C , Also E , Alias L , Caselles L , Gich I , et al. The developmental pattern of myotubes in spinal muscular atrophy indicates prenatal delay of muscle maturation. Journal of Neuropathology and Experimental Neurology 2009;68:474–481. 19525895 10.1097/NEN.0b013e3181a10ea1

[41] Mutsaers CA , Wishart TM , Lamont DJ , Riessland M , Schreml J , Comley LH , et al. Reversible molecular pathology of skeletal muscle in spinal muscular atrophy. Human Molecular Genetics 2011;20:4334–4344. 21840928 10.1093/hmg/ddr360

[42] Ploquin C , Chabi B , Fouret G , Vernus B , Feillet‐Coudray C , Coudray C , et al. Lack of myostatin alters intermyofibrillar mitochondria activity, unbalances redox status, and impairs tolerance to chronic repetitive contractions in muscle. American Journal of Physiology. Endocrinology and Metabolism 2012;302:E1000–E1008. 22318951 10.1152/ajpendo.00652.2011

[43] Mouisel E , Relizani K , Mille‐Hamard L , Denis R , Hourde C , Agbulut O , et al. Myostatin is a key mediator between energy metabolism and endurance capacity of skeletal muscle. American Journal of Physiology. Regulatory, Integrative and Comparative Physiology 2014;307:R444–R454. 10.1152/ajpregu.00377.2013 24965795

[44] Shorrock HK , Gillingwater TH , Groen EJN . Molecular mechanisms underlying sensory‐motor circuit dysfunction in SMA. Frontiers in Molecular Neuroscience 2019;12:59. 30886572 10.3389/fnmol.2019.00059 PMC6409332

[45] Fletcher EV , Simon CM , Pagiazitis JG , Chalif JI , Vukojicic A , Drobac E , et al. Reduced sensory synaptic excitation impairs motor neuron function via Kv2.1 in spinal muscular atrophy. Nature Neuroscience 2017;20:905–916. 28504671 10.1038/nn.4561 PMC5487291

[46] Augustin H , McGourty K , Steinert JR , Cocheme HM , Adcott J , Cabecinha M , et al. Myostatin‐like proteins regulate synaptic function and neuronal morphology. Development 2017;144:2445–2455. 28533206 10.1242/dev.152975 PMC5536874

[47] Elashry MI , Otto A , Matsakas A , El‐Morsy SE , Jones L , Anderson B , et al. Axon and muscle spindle hyperplasia in the myostatin null mouse. Journal of Anatomy 2011;218:173–184. 21208206 10.1111/j.1469-7580.2010.01327.x PMC3042751

[48] Lein ES , Hawrylycz MJ , Ao N , Ayres M , Bensinger A , Bernard A , et al. Genome‐wide atlas of gene expression in the adult mouse brain. Nature 2007;445:168–176. 17151600 10.1038/nature05453

[49] Wu HH , Ivkovic S , Murray RC , Jaramillo S , Lyons KM , Johnson JE , et al. Autoregulation of neurogenesis by GDF11. Neuron 2003;37:197–207. 12546816 10.1016/s0896-6273(02)01172-8

[50] Walker RG , Poggioli T , Katsimpardi L , Buchanan SM , Oh J , Wattrus S , et al. Biochemistry and biology of GDF11 and myostatin: similarities, differences, and questions for future investigation. Circulation Research 2016;118:1125–1141. 27034275 10.1161/CIRCRESAHA.116.308391 PMC4818972

[51] von Haehling S , Morley JE , Coats AJS , Anker SD . Ethical guidelines for publishing in the Journal of Cachexia, Sarcopenia and Muscle: update 2019. J Cachexia Sarcopenia Muscle 2019; 10: 1143‐1145. 31661195 10.1002/jcsm.12501 PMC6818444

